# DNA methylation mediated silencing of microRNA-145 is a potential prognostic marker in patients with lung adenocarcinoma

**DOI:** 10.1038/srep16901

**Published:** 2015-11-19

**Authors:** Wenjie Xia, Qiang Chen, Jie Wang, Qixing Mao, Gaochao Dong, Run Shi, YanYan Zheng, Lin Xu, Feng Jiang

**Affiliations:** 1 Department of Thoracic Surgery, Nanjing Medical University Affiliated Cancer Hospital, Jiangsu Key Laboratory of Molecular and Translational Cancer Research, Cancer Institute of Jiangsu Province, Baiziting 42, Nanjing, P.R. China, 210009; 2 The Fourth Clinical College of Nanjing Medical University, Nanjing 210000, China; 3 Department of Thoracic Surgery, Xuzhou Centre Hospital.

## Abstract

The molecular mechanism of down-regulated microRNA-145 (miR-145) expression in lung adenocarcinoma (LAC) remains largely unknown. We hypothesized that aberrant hyper-methylation of the CpG sites silenced the expression of miR-145 in LAC. In consideration of its pivotal role in LAC development and progression, we also evaluated the clinical utility of miR-145 as a prognostic marker. We assessed the DNA methylation status of the miR-145 promoter region in 20 pairs of LAC and the matched non-tumor specimens. We subsequently applied our own LAC tissue microarray containing 92 pairs of tumor and non-tumor tissues with long time follow-up records to evaluate whether miR-145 is a potential prognostic marker in LAC. The Sequenom EpiTYPER MassArray analysis showed that miR-145 was down-regulated in human LAC tissues accompanied by increased DNA methylation of its upstream region, which was further validated by the data from TCGA database. Significance was observed between miR-145 expression and clinic-pathologic parameters. Univariate and multivariate analysis revealed that miR-145 expression level was an independent risk factor for both OS and DFS in LAC patients. Taken together, DNA hyper-methylation in the miR-145 promoter region reduced its expression in LAC and miR-145 expression level might serve as a novel prognostic biomarker.

With approximately 1.5 million new cases diagnosed every year, lung cancer is the most prevalent cause of cancer deaths worldwide and lung adenocarcinoma (LAC) accounts for almost 40% of lung cancer deaths^1^,^2^. Already recognized as crucial factors in tumorigenesis, microRNAs (miRNAs) are proposed as promising biomarkers for early cancer detection and accurate prognosis as well as targets for more efficient treatment^3^,^4^,^5^,^6^. Recently, microRNA-145 (miR-145) was identified as a tumor-suppressive miRNA that was down-regulated in several cancer types, including prostate cancer, bladder cancer, colon cancer and ovarian cancer^7^,^8^,^9^,^10^,^11^,^12^.

Our previous study demonstrated that miR-145 was down-regulated in LAC tissues and miR-145 could suppress the proliferation of lung cancer-initiating cells (LCICs) derived from A549 *in vitro* by targeting Oct4 mRNA^13^. Our following study further validated that miR-145 could also modulate the epithelial-mesenchymal transition (EMT) properties of LAC cells by targeting Oct4^13^,^14^. By now, miR-145 was also reported to be involved in tumor progression by targeting c-Myc, AEG-1, EGFR, and NUDT1 in LAC^15^,^16^,^17^. Therefore, downstream mechanisms of miR-145 altering cellular processes are partially confirmed. In our present study we aim to explore the upstream molecular mechanism of down-regulated miR-145 expression in LAC.

It was suggested in recent studies that epigenetic alteration was involved in the dysregulation of miRNAs, especially, the hyper-methylation of CpG islands in the miRNA promoter region could lead to down-regulation of miRNAs in various cancers^18^,^19^,^20^. We hypothesized that aberrant hyper-methylation of the CpG sites in the miR-145 promoter region reduced its expression level in LAC. In the present study we carried out experiments in both LAC cell lines and the freshly resected LAC tissues to verify our hypothesis. And we also applied The Cancer Genome Atlas (TCGA) database to further validate our finding.

In view of its pivotal role in tumor development and progression, we also explored the clinical utility of miR-145 as a prognostic marker. We applied our own tissue microarray (TMA) containing 92 pairs of tumor and non-tumor tissues with long time follow-up records to investigate the association of miR-145 expression with clinic-pathological parameters, as well as the correlation between miR-145 expression and disease-free survival (DFS) and overall survival (OS) in LAC.

## Results

### Aberrant CpG methylation down-regulates the expression level of miR-145 in LAC

**Figure 1:**
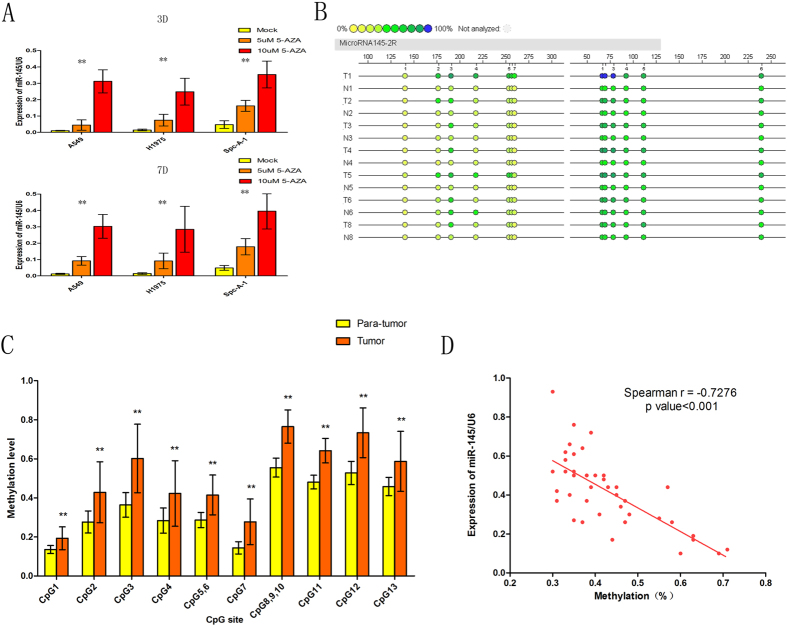
Aberrant hyper-methylation of the CpG sites silences the expression of miR-145 in LAC. (**A**) Real time-PCR demonstrated expression of miR-145 in three LAC cell lines after treatment with 5-aza-2-deoxycytidine for 3 or 7 days compared with mock-treated cells. Symbol (**) means a significant difference at P < 0.01. (**B**) Profiling of methylation levels of CpG sites in the miR-145 upstream region was presented as an epigram. (**C**) Average methylation level of each CpG site in para-tumor tissues and LAC tissues. Symbol (**) means a significant difference at P < 0.01. (**D**) Correlation analysis between mean methylation levels of the 300-upsteam region and corresponding miR-145 expressions.

As shown in Fig. 1A, pretreatment with 5-aza-CdR in LAC cell lines (A549, H1975 and Spc-A-1) for 3 or 7 days led to remarkably elevated miR-145 expression, indicating that there was a reverse correlation between DNA methylation and miR-145 expression. Subsequently, DNA methylation level of 13 CpG sites (which were divided into 10 CpG units) upstream of the miR-145 gene location were assessed using Sequenom EpiTYPER MassArray in 20 paired LAC and their matched para-tumor tissues. The results showed that all of the 13 CpG sites located upstream of miR-145 were hyper-methylated in tumors compared with matched para-tumor tissues (Fig. 1B,C and Table 1). Meanwhile we investigated the correlation between mean methylation levels and miR-145 expressions in LAC. Picking the methylation level as x-axis and miR-145 expression as y-axis, a significant negative correlation between the miR-145 expression and methylation levels was observed (Spearman r = −0.7272, p < 0.001; Fig. 1D). Taken together, these data indicated that DNA hyper-methylation in the upstream region of miR-145 might play an important role in the down-regulation of miR-145 in LAC.

### Data from TCGA database confirm that DNA methylation is a common mechanism of miR-145 down-regulation in LAC

**Figure 2:**
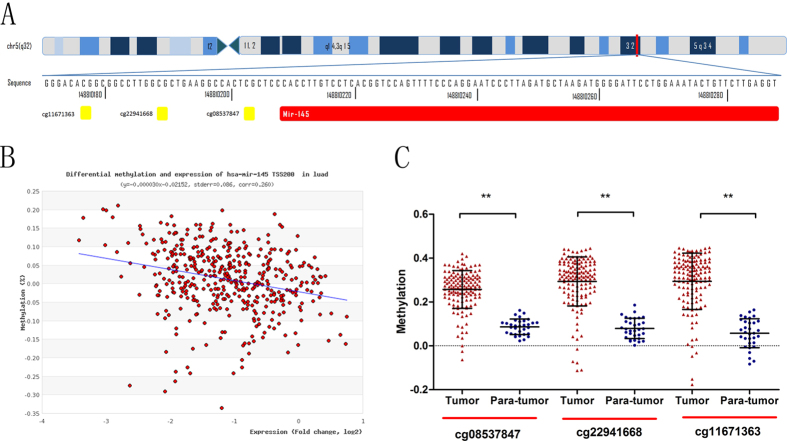
Data from TCGA database validates that miR-145 expression is associated with DNA methylation in human LAC. (**A**) Schematic representation of the location of miR-145 in chr5:148810209-148810296. Three CpG sites (cg11671363, cg22941668, cg08537847) included in TCGA database was represented as yellow plaques and analyzed in Fig. 2(B). (**B**) Expression of miR-145 was plotted against methylation level for three CpG sites (cg11671363, cg22941668, cg08537847). These data were extracted and plotted using a unique tool developed at Institute of Bioinformatics and Systems Biology at National Chiao Tung University (http://MethHC.mbc.nctu.edu.tw). MethHC revealed that expression of miR-145 was negatively correlated with DNA methylation of its 300-upstream region. (**C**) The gene chip data from Cancer Browser (https://genome-cancer.ucsc.edu/) validated that all of the three CpG sites were hyper-methylated in LAC tissues.

In consideration of the limited sample size of our study, we tried to dig into the newly available TCGA database for LAC to evaluate whether the depressed miR-145 expressions are in association with elevated DNA methylation. As depicted in Fig. 2A, miR-145 is located in chr5:148810209-148810296. TCGA database included methylation status of three CpG sites in the 5′ end of miR-145 (cg11671363, cg22941668, cg08537847), which are also parts of our assessed 13 CpG sites (Fig. 2A). MethHC (http://MethHC.mbc.nctu.edu.tw) revealed that miR-145 is commonly down-regulated in human LAC accompanied by increased DNA methylation of its 300-upstream region (Fig. 2B). Furthermore, we concretely compared the methylation levels of these three CpG sites between tumor and non-tumor tissues through the data downloaded from Cancer Browser (https://genome-cancer.ucsc.edu/), and the results showed that all of the three CpG sites are hyper-methylated in LAC tissues (Fig. 2C). Therefore, the data from our LAC samples, as well as external LAC cohort (n = 482) at TCGA database, strongly support the point that DNA methylation is a common mechanism of miR-145 down-regulation in LAC.

### miR-145 expression level might serve as a novel prognostic biomarker in LAC

**Figure 3:**
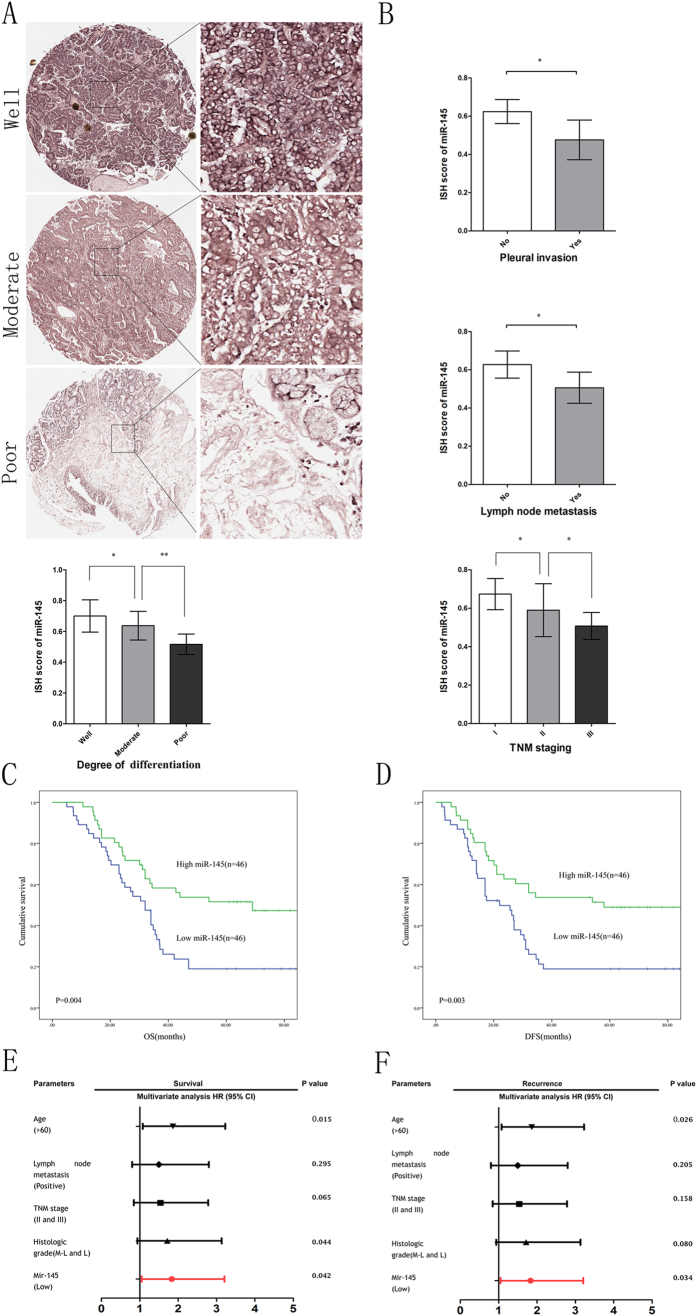
Down-regulated expression of miR-145 predicts poor prognosis in LAC patients. (**A**) Representative images of ISH staining of miR-145 in different differentiated LAC tissues from TMA. (**B**) Down-regulation of miR-145 in LAC was associated with clinic-pathological parameters (Pleural invasion, Lymph node metastasis and TNM staging). (**C**,**D**) The OS and DFS for the high and low miR-145 expression groups were analyzed by the two-sided long-rank test. (**E**,**F**) A multivariate analysis of the HRs showed that the down-regulation of miR-145 was an independent prognostic factor for the OS and DFS rates (by the Cox multivariate proportional hazard regression model). The HRs are presented as the means (95% CI). The variables included in the multivariate analysis were selected using a univariate analysis.

To further evaluate the clinical utility of miR-145 in the prognoses of LAC patients, we applied our own LAC tissue microarray containing 92 pairs of LAC and matched non-tumor tissues with long time follow-up records. Expression levels of miR-145 were detected by ISH with probes (Exiqon, Vedbaek, Denmark). As shown in Fig. 3A, we observed that well differentiated LACs showed higher miR-145 expression, as compared with those in poorly differentiated LACs samples. MiR-145 expression in the lymph node metastasis (stage N1-2) and pleural metastasis groups were remarkably lower than those in the non-metastasis groups. Lower miR-145 expression was also correlated with advanced TNM staging (P = 0.025, P = 0.015, P = 0.032, respectively, Fig. 3B). We designated the median miR-145 expression as a cutoff point, and the 92 LAC patients were divided into two groups according the miR-145 expression in tumors: a high miR-145 expression group (n = 46) and a low miR-145 expression group (n = 46). As shown in Table 2, correlation regression analysis indicated that expression of miR-145 was negatively correlated with differentiation (P = 0.011), lymph node metastasis (P = 0.003), pleural invasion (P = 0.035) and TNM staging (P = 0.029). OS and DFS were calculated by Kaplan-Meier analysis and log-rank test. As shown in Fig. 3C,D, patients with lower miR-145 expression exhibited poor OS (HR = 2.128, P = 0.004) and DFS (HR = 2.182, P = 0.003). The univariate and multivariate analysis further revealed that miR-145 expression level was an independent risk factor for both OS and DFS in LAC patients (Fig. 3E,F and Tables 3 and 4). The group with the lower expression of miR-145 also displayed shorter OS and DFS rates (OS: HR = 1.787, 95% CI = 1.021–3.128, p = 0.042; DFS: HR = 1.833, 95% CI = 1.048–3.206, p = 0.034). Collectively, these results indicated that miR-145 expression level might serve as a novel prognostic biomarker in LAC.

## Discussion

MiR-145 had been well described to be involved in tumor invasion and progression by targeting c-Myc, AEG-1, EGFR, NUDT1 and OCT4 in LAC^15^,^16^,^17^, and acknowledged to be a putative tumor-suppressor miRNA. The down-stream mechanisms of miR-145 altering cellular processes were partially confirmed in several published studies. In comparison, the upstream mechanism of which miR-145 was down-regulated in LAC still remained less well understood. Recently, epigenetic silencing of tumor-suppressor miRNA by aberrant DNA hyper-methylation has received increasing attention in a variety of cancers^21^. To explore the epigenetic mechanism regulating miR-145 in LAC, we compared the expression of miR-145 in three LAC cell lines before and after the treatment of 5-aza-CdR and found that de-methylation treatment induced remarkable miR-145 expression recovery, indicating hyper-methylation plays an important role in the regulation of miR-145 gene expression in LAC. In addition, we assessed the DNA methylation status of 13 CpG sites upstream of miR-145 using MassARRAY-based quantitative methylation analysis in 20 paired LAC and adjacent normal samples. As expected, the results revealed that miR-145 expression is commonly down-regulated in LAC tissues in association with increased DNA methylation, which was further confirmed in a large cohort from TCGA database. As far as we knew that miR-145 was firstly reported to be down-regulated owing to DNA methylation in LAC in our present study.

miRNAs are critical to both physiological and pathological processes, including tumor development and progression^22^,^23^,^24^. Recently, certain miRNAs are proved to be associated with clinical outcomes in colorectal cancer, pancreatic cancer, breast cancer, and HCC^25^,^26^,^27^,^28^. As a pluripotency repressor, miR-145 directly targets and silences OCT, SOX2 and KLF4 in human embryonic stem cells (ESC), which are required for self-renewal and pluripotency^29^. The overexpression of OCT4, SOX2, and KLF4 can reprogram or dedifferentiate somatic cells into induced pluripotent stem cells, which resemble the de-differentiation process that cancer cells undergo during tumor formation. Our previous studies also demonstrated that miR-145 suppressed the tumorigenesis and EMT process of LCICs by targeting Oct4^13^,^14^. Besides, down-regulation of miR-145 was also found to act on other tumor-associated targets such as c-Myc, AEG-1, EGFR, NUDT1 in LAC^15^,^16^,^17^. Down-regulated miRNA145 exhibited oncogenic or tumor suppressor properties by regulating multiple mRNAs, highlighting a powerful mechanism for the regulation of tumorigenesis in LAC. Importantly, the novel finding in our present study was that decreased miR-145 level correlated with poor differentiation, lymph node metastasis, pleural invasion, and advanced TNM staging in LAC. Moreover, Kaplan-Meier survival analysis and multivariate analysis revealed that reduced miR-145 level in LAC tumors was an independent predictor of shorter OS and DFS. Based on these results, miR-145 might serve as a potential prognostic marker during the follow up of LAC patients. The results also provided the possibility that miR-145 expression might be used to design optimal treatment for LAC patients, for instance, distinguishing locally advanced LAC patients who would benefit much from surgery.

In conclusion, our present study demonstrates that miR-145, which is regulated by DNA methylation, might be a valuable prognostic marker in LAC patients.

## Materials and Methods

### Patient samples and TMA

For methylation analysis, 22 freshly resected LAC specimens and their adjacent normal lung tissues were collected from the department of thoracic surgery, Cancer Institute of Jiangsu Province, China. The specimens were snap-frozen in liquid nitrogen.

TMA was performed to evaluate the clinical utility of miR-145 as a prognostic marker. Briefly, formalin-fixed paraffin-embedded archive tissues of 92 paired LAC and adjacent normal lung tissues were arranged in tissue array blocks (Shanghai Biochip Co., Ltd. Shanghai, China). Each tissue spot was accompanied with cases material including sex, age, pathologic type, pathologic grade and clinical stage. The study was in accordance with the provisions of Ethics Committee of Nanjing Medical University. A written informed consent was obtained from all participants involved in this study.

### Cell culture and treatment with 5-Aza-CdR

Human LAC cell lines A549, H1299 and SPC-A-1 purchased from American Type Culture Collection (ATCC,USA) were maintained in RPMI 1640 (Hyclone, Logan, UT) and supplemented with 10% fetal bovine serum (FBS, Invitrogen) at 37 °C in the humidified atmosphere with 5% CO_2_. After serum starvation for 24 hours, three LAC cancer cell lines were seeded in six-well plates, allowed to attach for 3, 7 days and treated with 0, 5 and 10 μM of 5-Aza-CdR, respectively.

### RNA extraction and RT-PCR

Total RNA was extracted from cell lines and tissues using mirVana RNRNA Isolation Kit (Applied Biosystems). Mature miR-145 levels were quantified using TaqMan microRNA Assay (Applied Biosystems). Quantification was normalized to the U6 small nuclear B non-coding RNRNA (RNRNU6B, Applied Biosystems), which served as an endogenous control. The ΔΔCT method was used to calculate the CT-value for each sample, and the results were expressed as 2^ΔΔCT^ to analyze the fold change (tumor vs. normal): ΔΔCT = (CT_target gene_ − CT_U6_) normal—(CT_target gene_ − CT_actin_) tumor.

### TCGA database validation

Data were downloaded from the TCGA portal and processed partly with the MethHC (http://MethHC.mbc.nctu.edu.tw) developed in the Institute of Bioinformatics and Systems Biology at National Chiao Tung University, Hsinchu, Taiwan. The discriminating methylation levels of the three CpG sites (cg08537847, cg22941668, cg11671363) in LAC and non-tumor tissues were analyzed by us on the basis of the gene chip data (TCGA_LUAD_hMethyl450) from Cancer Browser (https://genome-cancer.ucsc.edu/).

### DNA extraction and methylation analyses

Genomic DNA was extracted from 22 resected LAC specimens and their adjacent normal lung tissues using the genomic DNA rapid extraction kit (TIANGEN BIOTECH CO, LTD. Beijing, China). The quality and quantified were evaluated by gel electrophoresis and a NanoDrop spectrophotometer (GE Healthcare Life Sciences, Uppsala, Sweden), 2 of the 22 paired samples are not qualified. The genomic DNA from each sample was treated with sodium bisulfite using an EZ DNA methylation kit (Zymo Research, Orange, CA). The Sequenom MassARRAY platform (CapitalBio, Beijing, China) was used for quantitative analysis of miR-145 methylation. This system employs matrix-assisted laser-desorption/ionization time-of-flight mass spectrometry in combination with RNA base-specific cleavage. We used the following two pairs of primers on the basis of the reverse complemented strand of miR-145, (aggaagagagAGGTTAAGGTTTAGGGGTTAAGTGA, cagtaatacgactcactatagggagaaggctCTACATCCAACCCCATCTATAACAA) to amplify base pairs -354–(-62) upstream of miR-145, (aggaagagagGTTGTTATAGATGGGGTTGGATGTA, cagtaatacgactcactatagggagaaggctCTCTTACCTCCAAAAACAACCTTCT) to amplify base pairs -87- (+195) around miR-145. For each reverse primer, an additional T7 promoter tag (5′-cagtaatacgactcactatagggagaaggct-3′) for *in vitro* transcription was added. In addition, a 10-mer tag (5′-aggaagagag-3′) was added on each forward primer to adjust for melting-temperature differences between the forward and reverse primers. Altogether, 13 CpG sites were tested in this region. The spectra methylation ratios for each CpG site were generated by Epityper software version 1.0 (Sequenom).

### *In situ* hybridization (ISH) analysis

Expression of miR-145 in 92 pairs of LAC tissues were detected by ISH with probes for miR-145 (Exiqon, Vedbaek, Denmark). The 5′-3′ sequences (enhanced with LNA) were AGAACAGTATTTCCAGGAATCC with a DIG label at both the 5′ and 3′ ends. The TMA was in an oven at 60 °C for 60 mins then stored as slides overnight at 4 °C. Deparaffinized slides were in xylene and ethanol solutions at room temperature and then incubated with Proteinase-K for 7.5 mins at 37 °C. Slides were hybridized with 1000 nmol/L miR-145 probe in a hybridization buffer for 20 min at 50 °C then washed with SSC buffers. The remaining procedures were performed with a modified version of the manufacturer’s protocol. The intensity of miR-145 staining was scored by 0–2, according to the standards of 0–0.5 (weak staining), 0.5–1.5 (medium staining) and 1.5–2 (strong staining). Those expression scores were figured out by the intensities × the percentages of positive cells. In a blind method, individual specimens were evaluated by two pathologists, and those with scores of greater than 0.5 were defined as high expression, less than or equal to 0.5 were low expressions.

### Statistical analysis

All statistical analyses were performed using SPSS version 19.0 software (SPSS Inc., Chicago, USA). Student’s t-test and paired t-test were applied to evaluate methylation level of miR-145. A linear regression was performed to infer the correlation between the methylation percentage and expression of miR-145. The associations between clinical characteristics and miR-145 expressions were analyzed by the chi^2^ test. Survival curves were plotted using the Kaplan–Meier method, and differences between survival curves were tested using the log-rank test. Cox’s proportional hazards model was used to identify the factors that have significantly independent influence on survival. Statistical significance was set at P < 0.05.

## Additional Information

**How to cite this article**: Xia, W. *et al.* DNA methylation mediated silencing of microRNA-145 is a potential prognostic marker in patients with lung adenocarcinoma. *Sci. Rep.* **5**, 16901; doi: 10.1038/srep16901 (2015).

## Figures and Tables

**Table 1:** Comparison of methylation level between lung adenocarcinoma and adjacent normal tissue in each CpG site.

CpG sites	Number of pairs	Tissue types	Mean methylation level % (95% CI)
CpG1	20	para-tumor	13.55 (12.70–14.45)**
		lung adenocarcinoma tissue	19.30 (17.05–21.80)**
CpG2	20	para-tumor	27.65 (25.10–30.10)**
		lung adenocarcinoma tissue	42.85 (36.65–49.75)**
CpG3	20	para-tumor	36.40 (33.70–39.15)**
		lung adenocarcinoma tissue	60.20 (53.20–67.80)**
CpG4	20	para-tumor	28.40 (25.70–31.25)**
		lung adenocarcinoma tissue	42.25 (35.40–49.90)**
CpG5,6	20	para-tumor	28.65 (26.95–30.35)**
		lung adenocarcinoma tissue	41.50 (37.25–45.80)**
CpG7	20	para-tumor	14.40 (13.05–15.80)**
		lung adenocarcinoma tissue	27.75 (23.05–33.20)**
CpG8,9,10	20	para-tumor	55.50 (53.50–57.70)**
		lung adenocarcinoma tissue	76.50 (72.90–79.95)**
CpG11	20	para-tumor	48.10 (46.65–49.60)**
		lung adenocarcinoma tissue	64.20 (61.60–66.80)**
CpG12	20	para-tumor	52.75 (50.10–55.25)**
		lung adenocarcinoma tissue	73.35 (68.05–78.65)**
CpG13	20	para-tumor	45.80 (43.85–47.85)**
		lung adenocarcinoma tissue	58.70 (52.05–65.40)**

Symbol (**) means a significant difference at P < 0.01.

**Table 2:** Relationship between miR-145 expression and their clinic-pathologic parameters in 92 of NSCLC patients.

Variable	Cases (n)	Expression level of miR-145		P-value
		Low (n, %)	High (n, %)	
Ages				
< 60	51	26 (51.0)	25 (49.0)	0.834
≥ 60	41	20 (48.8)	21 (51.2)	
Sex				
Male	67	31 (6.3)	36 (53.7)	0.241
Female	25	15 (60.0)	10 (40.0)	
Differentiation				
Well	25	11 (44.0)	14 (56.0)	0.011*
Moderate	20	5 (25.0)	15 (75.0)	
Poor	47	30 (63.8)	17 (36.2)	
Tumor ize				
≤ 3cm	34	13 (38.2)	21 (61.8)	0.084
> 3cm	58	33 (56.9)	25 (43.1)	
Lymph node mtastasis				
No	50	18 (36.0)	32 (64.0)	0.003**
Yes	42	28 (66.7)	14 (33.3)	
Pleural invasion				
No	67	29 (43.3)	38 (56.7)	0.035*
Yes	25	17 (68)	8 (32)	
TNM staging				
I	34	11 (32.3)	23 (67.6)	0.029*
II	25	14 (56.0)	11 (44.0)	
III	33	21 (63.6)	12 (36.4)	

*P < 0.05 calculated with Pearson chi^2^ test.

**Table 3:** Univariate and multivariate Cox regression analyses of overall survival in 92 NSCLC patients following surgery.

Parameters	Univariate analysis			Multivariable analysis		
	HR	95% CI	P value	HR	95% CI	P value
Age ( > 60)	1.982	1.183–3.321	0.009	1.973	1.143–3.405	0.015
Gender (male)	1.050	0.591–1.865	0.867	—	—	—
Tumor diameter ( > 3m)	1.106	0.649–1.886	0.712	—	—	—
Pleural invasion (Positive)	1.602	0.933–2.750	0.087	—	—	—
Lymph node metastasis (Positive)	2.290	1.365–3.841	0.002	1.389	0.751–2.567	0.295
TNM stage (II and III)	2.364	1.412–3.957	0.001	1.787	1.021–3.128	0.065
Histologic grade (M-L and L)	2.595	1.470–4.580	0.001	1.832	1.015–3.308	0.044
miR-145 level (Low)	2.128	1.255–3.608	0.004	1.787	1.021–3.128	0.042

**Table 4:** Univariate and multivariate Cox regression analyses of disease-free survival in 92 NSCLC patients following surgery.

Parameters	Univariate analysis			Multivariable analysis		
	HR	95% CI	P value	HR	95% CI	P value
Age ( > 60)	1.917	1.144–3.213	0.014	1.863	1.076–3.226	0.026
Gender (male)	1.070	0.603–1.901	0.816	—	—	—
Tumor diameter ( > 3 m)	1.113	0.653–1.897	0.695	—	—	—
Pleural invasion (Positive)	1.631	0.950–2.800	0.076	—	—	—
Lymph node metastasis (Positive)	2.402	1.430–4.033	0.001	1.499	0.802–2.802	0.205
TNM stage (II and III)	2.286	1.367–3.823	0.002	1.536	0.847–2.785	0.158
Histologic grade (M-L and L)	2.499	1.415–4.411	0.002	1.714	0.937–3.135	0.080
miR-145 level (Low)	2.182	1.286–3.701	0.003	1.833	1.048–3.206	0.034
